# Complete Genome Sequence of a *Klebsiella pneumoniae* Strain Isolated from a Known Cotton Insect Boll Vector

**DOI:** 10.1128/genomeA.00850-14

**Published:** 2014-08-21

**Authors:** Enrique G. Medrano, Marissa M. Forray, Alois A. Bell

**Affiliations:** Insect Control and Cotton Disease Research Unit, U.S. Department of Agriculture, Agricultural Research Service, College Station, Texas, USA

## Abstract

*Klebsiella pneumoniae* (associated with bacterial pneumonia) was previously isolated from *Nezara viridula*, a significant vector of cotton boll-rot pathogens. We provide the first annotated genome sequence of the cotton opportunistic strain *K. pneumoniae* 5-1. This data provides guidance to study the bases of cotton pathogenesis by bacteria associated with vectors.

## GENOME ANNOUNCEMENT

*Klebsiella pneumoniae* causes bacterial pneumonia, and is a recognized source of nosocomial and community-acquired infections (1). An emerging problem in treatment of the bacteria is strain resistance to multiple antibiotics including those of the beta-lactamase family (1). Furthermore, although virulence factors have been identified, there is limited knowledge concerning the pathological pattern of infection. Notably, *Klebsiella* strains have been recovered from the southern green stink bug, *Nezara viridula* (2, 3), a known vector of cotton disease-causing agents (4). Potential correlations have been documented between increasing populations of *N. viridula* and seed necrosis. The strain sequenced here is capable of causing appreciable boll damage.

The sequenced *K. pneumoniae* subsp. *pneumoniae* strain Kp 5-1 (K3/K8 serotype) was isolated from *N. viridula* collected from a cotton field (3). A Roche 454 GS-Junior DNA analyzer was used to generate the draft genome sequence (16-fold coverage) of strain Kp 5-1. A Roche GS Titanium shotgun library was prepared, and two runs were pyrosequenced, yielding 42 Mb (208,251 reads with an average length of 317 bases) and 37 Mb (232,738 reads with an average length of 457 bases). Using the same genomic DNA stock, 3- and 8-kb paired-end Titanium libraries were constructed and pyrosequenced, producing 7 Mb (183,274 reads with 99,378 paired reads) and 42 Mb (20,765 reads with 16,641 paired reads), respectively. The genome was constructed using GS De Novo Assembler 454 version 2.7 and the CLC Genomics Workbench Linux platform, resulting in two scaffolds totaling 5.5 Mb. Both the largest scaffold (5.4 Mb) and an identified plasmid (186 kbp) contained contig gaps that were closed by cloning of PCR products and Sanger sequencing by employing an ABI PRISM 3100. Putative coding sequences were predicted by manual annotation using the NCBI BLAST program and the Prokaryotic Genome Annotation Pipeline program at the NCBI; both sets of results were manually curated.

In total, the genome contained 4,918 predicted coding sequences (CDSs), 6 rRNA operons, and 77 tRNAs. The annotation data revealed type IV and VI secretion systems, which are reportedly involved in plant disease in other bacteria (5) and potentially play a role in cotton infirmity. Sequencing the genome of strain Kp 5-1 will allow for the discovery of purported genes implicated in the pathogenesis of cotton bolls for the purpose of bypassing current traditional isolation and infectivity methods. This will allow for direct detection of the genes and their respective products involved in disease production.

### Nucleotide sequence accession numbers.

This complete genome sequence has been deposited at DDBJ/EMBL/GenBank under accession numbers CP008700 and CP008701.
